# Medico-Chirurgical Transactions, Vol. XVI. Part I

**Published:** 1831-01-01

**Authors:** 


**Medico-Chirurgical Transactions. Published by the Medical and Chirurgical Society of London. 8vo. pp. 235, Two Plates. London, 1830. Vol. XVI—Part I.**

The present volume or portion of a volume of the Society's Transactions, though not containing any paper of commanding importance, is far from barren in interesting cases. Amongst the contributors are, Dr. Babington, Mr. Lawrence, Dr. Bostock, Mr. Copland Hutchison, Mr. Langstaff, and other gentlemen, whose names are by no means unknown to fame. When men of erudition and eminence step forward to contribute their mites to the public stores of information, the aggregate must be important, though the items may be separately trivial. The substance of all useful books which issue from the press will always be found in the pages of this Journal. It has been our aim to separate the chaff as much as possible from the wheat, and to spread before our readers wholesome and esculent food, in preference to more fantastically cooked and more high-seasoned viands, which tickle the palate but tease the stomach. Side dishes are laid out on well ordered tables, but few there are who taste them.

In the present days, when medical books are a glut in the market, and medical booksellers are not acquiring rapid fortunes, the Transactions of Societies or the pages of the journals are the only vehicles in which prudential persons would choose to embark their facts or speculations. Like Aaron's rod indeed, the periodical press has almost engulphed its competitors within its insatiate jaws. The transactions of Societies scarcely pay the printing expenses; their circulation as books is very limited; and, as a natural consequence, they have lately become barren in articles of interest. It is the journals which swallow all, it is the journals that bring books, and hospitals, and lectures to the door of every surgeon in this mighty empire. There may be, nay, there is, individual evil in the exercise of the powers of the periodical press, but it cannot be denied that there is general good. Men might as well bid the sun stand still, or the tempest be lulled at their bidding, as attempt to stop the progress of that mighty engine. It has changed and is changing the face of Society, but whether for good or for evil, the great God, who rules all things, only can divine.

It is not our intention to enter on general speculations respecting the operation of periodical literature. It is enough for us, that we are constituent parts of the machinery, and humble agents in dispensing its benefits to mankind. It is not our business, it may not be our taste, to engage in political or party discussions, for we have ever thought that the most honorable part of our vocation was to convey information to those who are in need of it. In the best days of Rome, before Virtue had received her death blow in Utica, and the shadow of Liberty had blended with the mists of the night at Pharsalia, the proudest crown was won by him who had saved the life of a Roman. If at any time we have been the means of affording instruction where such was wanted, we should deem ourselves more than repaid for any labour we may have undergone. It is our avowed object to place all that is worth knowing in medicine, within the hands of those who wish to learn. And we can assure the younger portion of our readers that, when the difficulties and annoyances of practice crowd upon them, they will look back with pleasure on the moments which were spent in acquiring information; with regret and shame on those which were wasted in the perusal of ribaldry, and the encouragement of slander and slanderers. These remarks are not irrelevant to our present subject, for the inculcation of proper principles is never out of place.

In returning to the consideration of the Transactions before us, we may premise, before we enter on the review of particular articles, that those of minor bulk or of meaner interest will find a place in the Periscope department of this Journal. The more important will be noticed in the portion devoted to Reviews. Three papers are dedicated to cases of iliac aneurism. They occupy separate places in the volume, but we think it better to bring them under one view.

I. *Case of Aneurism of the External Iliac Artery, in which the Femoral Artery and Aorta were tied.* By J. H. JAMES, Esq. Surgeon to the Devon Hospital.

John Windsor, æt. 44, a spare but not unhealthy looking man, was admitted into the Devon Hospital, May 7th, 1829. He had been previously treated for diseased hip-joint, and for such Mr. James continued to treat him; but observing what he thought to be a glandular tumour above Poupart's ligament, he requested his colleague, Mr. Barnes, to look at it, and the latter ascertained it to be an aneurism. Part of the contents of the tumour could be expelled by pressure, but on that being withdrawn the blood returned with throbbing. The tumour continued to increase fast, and in June it occupied the whole of the iliac region, and projected considerably into the lower part of the abdomen. Mr. James determined to tie the artery below the tumour, notwithstanding there was no pulsation in the femoral artery in the groin. On the 2d of June, the operation was performed. Mr. James made an incision at a point exactly midway between the symphysis pubis and the spine of the ilium, reached the artery, which was seated at considerable depth, and tied it about half an inch below Poupart's ligament. In the evening there appeared, by measurement from the groin to the umbilicus, to be a diminution of three quarters of an inch in the bulk of the tumour. On the 4th, a little matter which had formed in the course of the ligature was let out, and the tumour was now diminished one inch. The temperature of the limb had been uniformly good. From this day the tumour began to increase, and on the 12th had equalled its former measurement; it began to point at its lower and outer part. On the 24th the integuments were grown tense and shining, and there was considerable pain; the man looked haggard and ill.

The tumour now increased fast in all its dimensions, especially at the part already mentioned, where the integuments soon became dusky red, and œdematous. The tumour was also growing towards the umbilicus. A consultation was called and it was determined to tie the aorta. This was done at half-past three, p. m., on the 5th of July, in the presence of the operator's colleagues and many medical gentlemen.

"The man was placed on the table with his shoulders slightly raised, the bowels having previously been thoroughly opened. I made the incision rather lower than in Sir A. Cooper's case, beginning it an inch above the umbilicus, and continuing it two inches below. I scratched through the linea alba below the umbilicus, and then proceeded to open the peritoneum nearly to the same extent as the external wound. This first part of the operation was somewhat impeded by very copious bleeding from the vessels of the integuments.

As soon as the division of the parietes was effected, the viscera protruded, and the efforts of the poor fellow continuing strong, I soon found myself embarrassed with almost the whole of the bowels: nearly all the colon, and a great part of the small intestines being pushed out, and presently quite distended with flatus, a circumstance frequently remarkable in the operation for strangulated hernia. I found the aorta without difficulty, pulsating strongly, but it was surrounded with dense cellular membrane, and a strong peritoneal covering was likewise interposed between my nail and it.

I may remark that even in the dead subject, it is sometimes a difficult matter to force the nail and finger between the aorta and the spine; in this case, embarrassed as I was by the coils of intestine, in which my hand was buried, it was particularly so. I enlarged the wound, but it was of little service; to have obtained sufficient room to push aside those inflated intestines would have required an incision of enormous extent; and supposing this made, there would hardly have been a probability of retaining them completely within the abdomen by any mode of suture during the exertions which the patient might make, and which it would probably be impossible to prevent.

I endeavoured cautiously to get the point of the aneurismal needle through, and succeeded; but when it reached the other side it broke at the handle, which in the one I had selected for its curve, was unfortunately of wood. I had little anticipated occasion for so much force. The broken part was so sharp that I was obliged to withdraw it, for fear of injuring the intestines. With some additional difficulty I got my finger, with Weiss's instrument upon it, under the artery; but even after this was effected, it was by no means easy, with the best assistance of my colleagues, to extricate the short needle bearing the ligature, so much did the intestines interfere with every kind of manipulation. When the ligature was underneath, I kept the intestines out of the way with the fingers of both my hands, and placed one of my thumbs on the vessel, whilst Mr. Luscombe drew it, first on my thumb, and then on the artery; by this I prevented any thing from being included, a caution which Sir A. Cooper has particularly dwelt upon. The ligature was then drawn tight, and the tumour became flaccid; at the same time the patient complained of deadness in the lower extremities. The ligature was cut close.

From the tension of the muscles and the inflated state of the intestines, they were not easily returned; but when they had been replaced, five needles were passed through the integuments, and the wound having been secured perfectly by the quill suture, large straps and a bandage were added, and the man was put to bed." 9.

During the operation he suffered much and was at times very faint, but was revived by brandy and water, with laudanum in it. He complained of great pain in both the lower limbs, which on the aneurismal side soon amounted to agony, and tortured him till he died, which was at 7, p. m., on the same evening. Till the cold dews of death were settling on this unfortunate patient, the temperature of the lower limbs continued the same as the trunk.

*Dissection.* The tumour was considerably collapsed, and the discoloration on its surface gone. The wound measured four inches. A large quantity of blood, partly coagulated, was found in the abdomen amongst the intestines. This may have proceeded from the vessels of the parietes, which is unlikely; or from a small branch, observed at the time of the operation to be wounded, in one of the underfolds of the mesentery. The intestines were still distended with air, and apparently uninjured. The ligature was firmly drawn round the aorta, five lines below the inferior mesenteric artery, and eleven above the bifurcation of the common iliacs; it was about an inch below the duodenum. Some very dense cellular membrane, and a small vein which was traced along the coats of the aorta, and terminated in the inferior mesenteric, were included in the ligature. The vena cava was uninjured.

The aneurismal tumour was of enormous size, extending, even when collapsed, from the upper part of the thigh to the side of the vertebral column, and filling the whole of the ilium. It projected on one side far into the pelvis; on the other, it occupied the inferior posterior part of the abdomen. The ilium on which it rested was bare, scabrous, and absorbed nearly through to the acetabulum; hardly any of the pubes on that side, as far as the symphysis, was left. The walls of the outer and lower part of the tumour, where sloughing had been threatened, were very thin and formed only of attenuated muscular fibres and skin; the blood here was grumous and fluid. The sac elsewhere was thick and dense, and the peritoneum adhered most firmly to its anterior surface. Let us glance at the state of the vessels. The division of the aorta into the common iliacs was natural; so was the common iliac on the left side. The left internal iliac was natural. The external iliac was immediately implicated in the tumour, along the anterior surface of which it ran broad and flat, about two thirds of its length, at which point it was abruptly lost. On opening the sac no traces of the posterior part of the artery could be found, but an inch and a half lower down it was recognized again, forming a pouch from which proceeded the femoral arteries. The sac was chiefly filled with concentric lamellæ of organized fibrine: it also contained much coagulated and some grumous blood. There were some diseased spots in the aorta; the lower part of the external iliac, forming the pouch before mentioned, was thickened and diseased in the highest degree.

The following circumstances were observed in the arteries below the sac. The external iliac did not terminate, as usual, in the common femoral, but gave off two trunks of nearly equal size; from the inner, corresponding with the profunda, the epigastric arose. The ligature had been applied to the superficial femoral, half an inch or more below the level of the origin of the epigastric. Above the ligature was a perfect clot, reaching as high as the external iliac; below it the remains of a clot which had been disturbed by a probe. At the point of ligature the artery was obliterated. A large quantity of dense lymph was deposited externally, restoring the apparent size of the artery, and burying the noose of the ligature firmly within it.

In the aorta the clots were imperfect both above and below. There was no appearance of disease in the hip-joint. No mention is made of the state of the heart and great vessels in the thorax, which we cannot but deplore.

Mr. James laments that he did not discover the aneurism sooner, yet he feels assured that, had he done so, he would have tied the artery below the tumour. If there were any room whatever for the application of a ligature to the external iliac artery above the tumour, we should hold Mr. James, or any other surgeon, most culpable in tying the vessel below it. Experience has proved that, whatever be the merits or demerits of this operation, it is not to be compared in point of security with the Hunterian. The ligature of the external iliac artery has ever been considered more successful than that of the superficial femoral; at all events, it is not a dangerous operation. But the operation below the tumour, for iliac aneurism, has proved singularly unfortunate, and the origin of the epigastric and circumflexa ilii branches, between the sac and the ligature, appear to be explanatory of the circumstance. If then no space is afforded for operating on the external iliac, it comes to be a question whether the surgeon should operate below the tumour, or tie the common iliac. For our own parts, we must say that, under ordinary circumstances, we should be inclined to try the former and the milder method, though our hopes of success would be far from sanguine. This operation is by some very oddly considered a party question. The radicals are furious if a man presumes to question the wonderful success they pretend to attribute to it. Probably there is no operation in modern times, respecting which so many and such unblushing falsehoods have been promulgated by its advocates.

The operation on the abdominal aorta could hardly have been undertaken in the present case, with the slightest hopes of success. We confess that we look with some degree of aversion, on operations performed without fair probabilities of a fortunate issue. Unsuccessful operations, especially if they are severe, create a prejudice against surgery in the public mind. Surgeons are either looked upon as ignorant of the chances which their measures offer to those who submit to them, or suspected of an overweening and disgusting fondness for the knife, at the expense of the blood and the sufferings of their patients. Suspicion in such a case is as terrible as certainty, and every unfortunate operation that occurs will probably deter persons from undergoing others that would prove successful. This is a consideration which should weigh with all humane and reflecting men.

Mr. James is much surprised at the violent pain experienced in the lower extremities of the patient on tightening the ligature round the aorta. By being confined to the lower extremities it depended evidently on the vessels supplying them, and as neither the vein nor a nerve were included, it follows that the cause must be searched for in the artery. It is well known that some old persons, in whom the arterial tunics are more or less diseased, and the circulation consequently feeble in the lower limbs, are subject to ulcers or sloughing on the leg, attended with excessive pain. Such cases are modifications of the gangrena senilis and run into it; the pain is excruciating, and only relieved, if relievable at all, by the free administration of opium. There is a man at present in St. George's Hospital, with such a sloughy ulcer on the calf of the leg. Almost all his arteries appear to be rigid. There was another patient in the same hospital, for whom the toe was amputated in consequence of caries and dreadful pain; he had long been paralytic on the same side. After the amputation, a creeping, chronic sloughing was set up, which had not extended higher than the foot in the course of several months. The pain in the leg and foot were most excruciating, the poor man often shrieking out with agony. The arteries in this man were extensively diseased, the circulation remarkably torpid. When the abdominal aorta is suddenly tied the effect may be said to be an extreme degree of this same affection, and in the case of Mr. James the arteries were diseased to boot. We are therefore inclined to view the agonizing pain in the lower limbs, as dependent, in that case, on the sudden arrest which their circulating system received; in short, on the ligature of the great artery, and on that alone.

We now pass to the case of Mr. Crampton.

II. *Case of Aneurism of the External Iliac Artery, in which a Ligature was applied to the Common Iliac Artery.* By PHILIP CRAMPTON, M. D. F.R.S. &c. &c. &c.

The following sentiment, with which Mr. Crampton opens his paper, is just.

"To the practical surgeon it will lose nothing of its interest, either because it is not the first, or because it has not been successful. In fact, paradoxical as it may appear, the progress of surgery is more likely to be advanced by a faithful account of unsuccessful than of successful cases. In the successful cases nature performs her work in secret, and often leaves us at a loss to determine whether the success which we witness is to be attributed to the efforts of art, or to the unassisted resources of nature. In the unsuccessful cases, on the contrary, we have usually an opportunity of examining after death the effects of our operations, we see the results of those efforts which in concurrence with or in opposition to ours, nature makes for her relief, and are thus enabled to discriminate between the means which are likely to advance, and those which are likely to retard her work. I am further induced to submit this case to the consideration of the surgical profession, because in the operation I adopted a proceeding different from Mr. Mott's, and one which renders the tying of the Common Iliac Artery, or even of the Abdominal Aorta itself, *(so far as the operation itself is concerned,)* comparatively easy and safe."

Unsuccessful cases teach us the morbid anatomy of operations, and the same necessity which exists for the study of the morbid anatomy of diseases, must also exist for the study of it here. But as morbid anatomy in general is not all in all, neither is it to be looked on as our polar star in the science of surgery. Fatal cases are advantageous only, in teaching us to prevent others from becoming so. We must study cases which end well with at least as much diligence as those that end ill, or we shall grow mere morbid anatomists.

*Case.* Private P. M'Gowan, 8th Foot, aged 30, hitherto healthy, was admitted into the General Hospital, Phœnix Park, Dublin, on the 8th of July, 1828, under the care of Mr. Crampton. There was a pulsating tumour, extending from about three inches below the crural arch on the right side to within three inches of the umbilicus. It was divided by a furrow in the line of Poupart's ligament into two portions. The lower or femoral was scarcely compressible, and, though throbbing strongly, had not the peculiar aneurismal thrill. The upper portion formed an obvious swelling in the right hypogastric region, was soft and compressible as if filled with fluid blood, and at a particular point, three inches above the crural arch, the aneurismal thrill was unusually distinct. There was also a pulsating and compressible tumour, about the size of a pullet's egg, in the right ham. The man complained of great pain in the thigh and leg; pulse 110, full and throbbing; tongue white; little appetite.

He attributed the disease to a fall in wrestling, received about nine months previously. He felt little pain at the time, but two days afterwards he observed a small pulsating tumour "at the rim of his belly." He continued at his duty till the 20th of May. During the last fortnight the tumour had rapidly increased, and the pain was so severe that he was willing to submit to any operation. He was bled, purged, put on fever diet, and took tincture of digitalis. The blood for the first four bleedings was highly buffed and cupped, on the 5th it had a natural appearance. On the 18th of July he was considered in a proper state for the operation, which was performed in the presence of Professors Colles, Macartney, and Wilmot, Mr. Stringer, (the surgeon of the hospital,) Dr. Ramsay of Dundee, and other gentlemen. The steps of the operation must in justice be given in the words of the distinguished operator and author.

" The first incision commenced at the anterior extremity of the last false rib, proceeding directly downwards to the os ilium, it followed the line of the crista ilii, keeping a very little within its inner margin, until it terminated at the superior anterior spinous process of that bone, the incision was therefore chiefly curvilinear, the concavity looking towards the navel. The abdominal muscles were then divided to the extent of about an inch, close to the superior anterior spinous process, down to the peritoneum ; into this wound, the fore finger of the left hand was introduced, and passed slowly and cautiously along the line of the crista ilii, separating the peritoneum from the fascia iliaca, the peritoneum touching the fore-part, and the fascia iliaca the back-part of the finger. A probe-pointed bistoury was now passed along the finger to its extremity, and by raising the heel of the knife, while its point rested firmly on the end of the finger as on a fulcrum, the abdominal muscles were separated from their attachments to the crista ilii by a single stroke. By repeating this manœuvre, the wound was prolonged until sufficient room was obtained to pass down the hand between the peritoneum and the fascia iliaca. Detaching the very slight connections which these parts have with each other, I was able to raise up the peritoneal sack with its contained intestines on the palm of my hand, from the psoas magnus and iliacus internus muscles, and thus obtain a distinct view of all the important parts beneath ; and assuredly a more striking view has seldom been presented to the eye of the surgeon ; the parts were unobscured by a single drop of blood ; there lay the great Iliac Artery, nearly as large as my finger, beating awfully at the rate of 120 in a minute, its yellowish white coat contrasting strongly with the dark blue of the Iliac Vein which lay beside it, and seemed nearly double its size ; the ureter in its course to the bladder lay like a white tape across the artery, but in the process of separating the peritoneum, it was raised from it with that membrane to which it remained attached. The fulness of the Iliac Vein seemed to vary from time to time, now appearing to rise above the level of the artery, and now to subside below it. Nothing could be more easy than to pass a ligature round an artery so situated. The fore finger of the left hand was passed under the artery, which with a little management was easily separated from the vein ; and on the finger, (which served as a guide,) a common eyed probe furnished with a ligature of moistened catgut was passed under the vessel. A surgeon's knot was made in the ligature, and the noose gradually closed, until Mr. Colles, who held his hand pressed upon the tumour, announced that 'all pulsation had ceased !' A second knot was then made, and one end of the ligature cut off short. On examining the vessel after it had been tied, it was found to be full, and throbbing above the ligature, but empty and motionless below it. The external wound was united by three or four points of suture, and supported by long straps of adhesive plaster. The operation was completed in twenty-two minutes ; the patient, who was a firm minded man, made no complaint during the operation, not even when the ligature was closed upon the artery. The tumour, immediately after the operation, was diminished nearly one-third, the diminution being confined to the abdominal portion ; ten minutes after the operation, the pulse was 96 ; at 7 p. m. Mr. Stringer, finding the pulse full and bounding, took 20 ounces of blood from the arm; at 10 p. m. I found him tranquil, no pain, pulse 88, the limb, with the exception of the toes, warm: Saphena Vein full; additional flannel was wrapped round the foot." 163.

Next day the toes were not quite so warm as those of the other foot. The bowels not being open, he was ordered castor oil and an enema if required. These were not sufficient, but calomel, with castor oil, and an enema of turpentine were effective on the 20th. In fifty hours after the operation, Mr. Corr, a house pupil, remarked that there was an obscure pulsation in the tumour. On the 21st, the pulsation in the whole tumour was decided, but there was no thrill. There was neither pulsation in the femoral artery nor in the aneurism at the ham, which was reduced to half its original bulk. The pulse was 88. The thermometer at the groin stood at 98, at the hams 97, at the ankles 94, at the ball of the *left* great toe 87, at the right 88. On the 22d the pulsation was thought to be accompanied with an obscure thrill, and on the 24th the pulsation in the abdominal portion of the tumour was rather more distinct. He was bled in the erect posture to deliquium, had fever diet, and 20 drops of tinct. digital. every third hour. On the 25th the pulsation was more distinct, the thrill quite perceptible; there was no pulsation in the femoral artery. The blood drawn was buffed and cupped, and 12 ozs. more were abstracted. The quantity of digitalis was increased to 30 drops; fever diet. The ligature came away in the night and was lost in the bed. Whilst turning in bed on the 26th, he suddenly felt a severe pain in the thigh and knee, as if the knee was tearing off him; and the fore part of the thigh was all numb. In ten minutes the pain subsided. From the early return of the pulsation, with so much more strength than could be expected if the tumour were supplied by anastomosis only, it was concluded that the catgut ligature had become macerated and given way, a supposition that proved but too well founded. On the 28th he appeared better, and the wound was nearly healed. At 6, p. m. whilst sitting in his bed, the blood suddenly gushed from the wound in a torrent, he fell backwards, and in less than a minute he expired. On the following day the body was examined in the presence of the gentlemen who had witnessed the operation.

*Dissection.* "The intestines being removed, the peritoneum raised, and the great abdominal vessels laid bare, the common iliac artery, at about three-fourths of an inch from the bifurcation of the aorta, was lost in an oblong tumour, about three-fourths of an inch in diameter, and one and a half in length; the tumour terminated upon, but did not communicate with, the aneurismal sac. On cutting into the tumour, about half an ounce of greenish pus flowed from the wound and discovered the artery, which appeared somewhat contracted at one part, and its coats deeply indented, but not cut through, marking the place where the ligature had been applied. On blowing into the iliac artery from above, bubbles of air escaped freely from the external wound from whence the blood had issued; water injected by a syringe escaped by the same passage; clearly establishing the important fact, that the ligature, which was of cat-gut, had been dissolved by the heat and moisture of the wound, and thrown off, before the obstruction of the artery, or the coagulation of the blood in the aneurismal sac, had been completed. It further appeared that the dissolution of the ligature had caused a small abscess to form in the place which it occupied. On slitting up the artery, the internal and middle coats were found to be completely divided in the whole circumference of the vessel, and small portions of lymph adhered to its internal surface. The popliteal aneurism was far advanced towards a cure; the contents of the sac were quite solid, and the tumour was reduced to about the size of a walnut: the artery, for six inches above the sac, was filled with a firm coagulum." 168.

Mr. Crampton concludes, from this case, that the operation of tying the common iliac artery is not only feasible, but easy. The difficulties encountered by Valentine Mott were occasioned by his having begun his incision *too low*. This brought him in contact with the aneurismal tumour, from which he was obliged, with great labour and risk, to detach the peritoneum; then he had the whole mass of the tumour between him and the artery which he was to tie; and, lastly, he had the intestines pressing down upon him, and producing such a complication of difficulties as few men, except himself, could have surmounted. Mr. Crampton began his incision where Mr. Mott's terminated. Mr. Crampton does not pretend to decide if the operation of tying the aorta can ever be justifiable. But if such an operation should ever be determined on, he believes it could be readily accomplished by such a proceeding as that which he has now described.

It is impossible not to be struck with the contrast between an operation so truly surgical and scientific as this of Mr. Crampton's, and the one adopted by Mr. James. It is difficult to pronounce, that the operation of tying the common iliac can never be necessary; but of course no surgeon would resort to such a step, if, by any possibility, the external iliac could be secured in a part not implicated in the aneurismal tumour below. The operation is severe in every point of view, all good and careful surgeons would shun it if they could, but no man, we repeat, can venture to affirm that it might not, on particular occasions, be successful as well as justifiable.

It cannot but prove a source of regret, that so able an operator and scientific a gentleman as Mr. Crampton should have been betrayed into using a catgut ligature on such an occasion. We had thought that the varied and numerous experiments on catgut, fisherman's silk, and so forth, had left a firm conviction, in the minds of the profession, that a single silk ligature was most to be trusted. At all events, in performing so great an operation, Mr. Crampton should not have experimented on ligatures. He knew what single silk would do, but he did not know, and on such an occasion should hardly have tried, what catgut would not. He did try, the catgut rotted, and his patient was lost.

III. *An Account of the Dissection of the Parts concerned in the Aneurism, for the Cure of which Dr. Stevens tied the internal Iliac Artery at Santa Cruz, in* 1812. By Mr. Richard Owen, Surgeon.

Our readers will no doubt remember, that the internal iliac artery was said to be tied by Dr. Stevens for glutæal aneurism. The case was published in the Medico-Chirurgical Transactions, vol. V. p. 422. The patient was Maila, a negro woman. The disease had commenced nine months previously, and the operation was performed by Dr. Stevens on the 27th of December, 1812. In six weeks the woman was perfectly well, and she enjoyed good health for 10 years, when she died of an affection of the chest, in 1822. Dr. Stevens examined the parts within the pelvis in the presence of Dr. Kerr, and other medical gentlemen of Santa Cruz. He ascertained, by injection, that the internal iliac artery had become impervious at the part where the ligature had been applied: and he found that the ischiatic artery was continued, in the character of a ligamentous chord, to the place of its exit from the pelvis. The glutæal artery was pervious at its origin.

On Dr. Steven's arrival in this country, he deposited the preparation in the Museum of the Royal College of Surgeons, and the dissection was intrusted to Mr. Richard Owen. The result of his investigations is now published, and all must feel an interest in learning the particulars.

As a preparatory step, Mr. Owen threw in some fine injection by the profunda; it ran out freely at the opening which had been made in the origin of the glutæal. The external and internal iliac arteries were given off in the usual manner. The left internal iliac had become obliterated just below the origin of the ilio-lumbalis. In the state of a ligamentous cord, it descended towards the ischiatic notch for the space of an inch, and then, suddenly resuming its natural diameter, it again became pervious, and so continued for the space of half an inch. The glutæal artery arose from the lower part of this space; a sacro-lateral vessel from about the middle; and the obturator artery from the upper part of it. The latter vessel was entirely obliterated; but the sacro-lateral was pervious, of the size of a crow-quill, and passed inwards to the second sacral foramen. The glutæal artery, of its natural size, received close to its origin two vessels, as large as the preceding, given off from the sacro-lateral artery, near the third and fourth sacral foramina of the left side. The anastomoses of the sacro-lateral with each other and the sacro-median were large and tortuous. Immediately after the origin of the glutæal artery, the ischiatic, obliterated and cord-like, passed on to the lower part of the ischiatic notch.

The glutæal artery was in a healthy condition, and of the natural size. An elongated tumour, situated between the tuberosity of the ischium and the great trochanter, indicated the true seat of the original disease. This tumour, in length three inches and a half, and in breadth two thirds of an inch, was of that branch of the sciatic artery which seems to form its continuation, and accompanies the nerve. It consisted of layers of condensed cellular membrane, and the peculiar fibrous arterial coat. It contained a quantity of dark-coloured granular, not lamellated, coagulum, which, when removed, discovered the internal surface of the sac somewhat irregular, and raised in small patches by the deposition of soft matter. In some places, it appeared to retain the smooth character of the arterial lining membrane. From the ischiatic notch to the tumour, the artery was completely choked up, its texture altered, and the remains of its cavity filled with indurated and partly calcareous matter. From the lower part of the tumour, the sciatic artery was continued down the posterior part of the thigh, nearly as large as the femoral in front. Its cavity was not proportionally great, for its coats were at least one-half thicker than any artery of the same size with itself. It was obliterated for about the space of an inch below the sac, and became pervious after receiving an anastomosing vessel from the arteria profunda.

A vessel ramifying between the glutæus maximus and medius, and distributing branches to these muscles, was connected with the commencement of the sac, from which it had probably arisen. It did not, however, open into it, but, becoming contracted near the point of attachment, it there gave off a small artery to the quadratus femoris, and received its blood by anastomosing, near the crista ilii, with a superficial branch of the glutæal. A smaller vessel was similarly attached to the lower part of the sac, but did not communicate with it; for the blood which it received from branches ramifying in the neighbourhood, was diverted from the sac by a small branch, given off at the point of attachment.

Sir A. Cooper examined the preparation, and having himself laid open the remains of the sac, felt perfectly satisfied of the existence of the aneurism and obliteration of the internal iliac artery by Dr. Stevens' operation.

The foregoing is a very interesting dissection, and science, as well as justice to Dr. Stevens, demand that the public should possess the particulars. We know that doubts have been entertained respecting the performance of the operation by Dr. Stevens, and, without questioning the propriety of scepticism in such a case, we are happy that the preparation has stamped the truth of the narration of the operator. It is evident that Dr. Stevens was mistaken in supposing that the aneurism arose from the glutæal artery; it sprung from the ischiatic. It is remarkable that, after the lapse of ten years, loose coagula should be found in the sac. The cure was not effected by the complete arrest of the circulation through the artery. Probably this seldom happens. The ligature having been applied on the internal iliac, below the ilio-lumbar, the blood got round into the trunk through the medium of the sacra-media and sacro-lateral branches. The glutæal was thus supplied and the ischiatic, but the current in the latter was effectually stemmed, and its cavity became obliterated to the distance of an inch below the sac. Now it is singular that the blood should coagulate in the ischiatic artery and not in the glutæal, although both were placed in very similar circumstances, quoad the circulation. The glutæal, however, received more immediately the blood from the sacro-lateral branches. The circumstance of the internal iliac becoming pervious below the ligature, is not much to be wondered at, for it is not uncommon for the femoral artery to be pervious between the ligature and the aneurismal sac, although the latter shall become obliterated.

Besides this operation of Dr. Stevens', the internal iliac artery is said to have been tied four times. The second operation, reckoning that by Dr. S. as the first, was performed by Mr. Atkinson, at the York County Hospital. The patient died nineteen days after the operation. An analysis of the case is given in Cooper's Surgical Dictionary. It is not clear whether the disease was in the glutæal or ischiatic. The third case is said to have been successful—the operator, an army surgeon in Russia, on whom the Emperor Alexander settled a pension. The fourth operation was performed by Mr. Thomas, of Barbadoes. The patient died, and the preparation has been deposited by Sir Astley Cooper in the Museum of Guy's Hospital. The fifth and last case is related in the American Journal of the Medical Sciences, for February, 1828. It was successful, and the operator was S. Pomefroy White, surgeon, Hudson, New York. Dr. Stevens remarks that Mr. White's character is unimpeached in his native country.

With the enumeration of these cases, we take leave, for the present, of the operation of tying the internal iliac. If a case should present itself in which it should be deemed justifiable, our readers may perceive what the real or imputed success of their predecessors has been in its performance.

